# Intensified antineoplastic effect by combining an HDAC-inhibitor, an mTOR-inhibitor and low dosed interferon alpha in prostate cancer cells

**DOI:** 10.1111/jcmm.12583

**Published:** 2015-03-26

**Authors:** Igor Tsaur, Lukasz Hudak, Jasmina Makarević, Eva Juengel, Jens Mani, Hendrik Borgmann, Kilian M Gust, David Schilling, Georg Bartsch, Karen Nelson, Axel Haferkamp, Roman A Blaheta

**Affiliations:** a Department of Urology, Johann Wolfgang Goethe-UniversityFrankfurt am Main, Germany; b Department of Vascular and Endovascular Surgery, Johann Wolfgang Goethe-UniversityFrankfurt am Main, Germany

**Keywords:** prostate cancer, combination therapy, valproic acid, everolimus, interferon alpha

## Abstract

A significant proportion of men diagnosed with prostate cancer (PCa) eventually develop metastatic disease, which progresses to castration resistance, despite initial response to androgen deprivation. As anticancer therapy has become increasingly effective, acquired drug resistance has emerged, limiting efficacy. Combination treatment, utilizing different drug classes, exemplifies a possible strategy to foil resistance development. The effects of the triple application of the histone deacetylase (HDAC) inhibitor valproic acid (VPA), the mammalian target of rapamycin inhibitor everolimus and low dosed interferon alpha (IFNα) on PCa cell growth and dissemination capacity were investigated. For that purpose, the human PCa cell lines, PC-3, DU-145 and LNCaP were treated with the combined regimen or separate single agents. Cell growth was investigated by the MTT dye reduction assay. Flow cytometry served to analyse cell cycle progression. Adhesion to vascular endothelium or immobilized collagen, fibronectin and laminin was quantified. Migration and invasion characteristics were determined by the modified Boyden chamber assay. Integrin α and β subtypes were investigated by flow cytometry, western blotting and RT-PCR. Integrin related signalling, Epidermal Growth Factor Receptor (EGFr), Akt, p70S6kinase and extracellular signal-regulated kinases (ERK)1/2 activation were also assessed. The triple application of VPA, everolimus and low dosed IFNα blocked tumour cell growth and dissemination significantly better than any agent alone. Antitumour effects were associated with pronounced alteration in the cell cycle machinery, intracellular signalling and integrin expression profile. Combining VPA, everolimus and low dosed IFNα might be a promising option to counteract resistance development and improve outcome in PCa patients.

## Introduction

Currently, prostate cancer (PCa) is the most frequently occurring tumour and the third most common cause of cancer mortality in European men 1. PCa commands significant clinical and research activity, which has resulted in substantial therapeutic progress in disease management in the last years 2. Risk adapted approaches incorporating personal life expectancy, comorbidities and treatment preference are increasingly considered for localized PCa offering patients a choice of radical prostatectomy, radiation therapy or active surveillance 3. Unfortunately, up to 40% of men diagnosed with PCa eventually develop metastatic disease, inevitably progressing to castration resistance despite an initial response to medical or surgical castration 4. For this advanced disease stage, several new hormonal, immuno- and chemotherapeutic agents as well as radiopharmaceuticals have resulted in improved overall survival in randomized phase 3 trials, receiving recent approval for clinical application 5. Despite these advances, median survival with first-line therapy of metastatic castration-resistant PCa is about 20 months and with post-docetaxel therapy approximately 15 months 6. Therefore, the need to extend and improve established treatment options persists.

Unfortunately, as anticancer therapy becomes increasingly effective, acquired drug resistance materializes 7. A possible strategy to foil resistance development involves combined therapy, utilizing different drug classes 8. Evidence has been provided showing enhanced activity of the histone deacetylase (HDAC)-inhibitor valproic acid (VPA) combined with low dosed interferon alpha (IFNα), compared to monotherapy in PCa cells 9. Antineoplastic effects of HDAC-inhibition have also been augmented when combined with the mammalian target of rapamycin (mTOR) inhibitor everolimus 10,11.

The current study was designed to investigate the effects of a triple therapy consisting of IFNα, VPA and everolimus on PCa cell growth and dissemination, compared to effects due to single agents.

## Materials and methods

### Cell cultures

Human prostate tumour cell lines PC-3, DU-145 and LNCaP were obtained from DSMZ (Braunschweig, Germany). Tumour cells were grown and subcultured in RPMI 1640 (Gibco/Invitrogen; Karlsruhe, Germany). The medium contained 10% foetal calf serum (FCS), 2% 2-(4-(2-Hydroxyethyl)-1-piperazinyl)-ethansulfonsäure HEPES-buffer (1 M, pH 7.4), 2% glutamine and 1% penicillin/streptomycin. Subcultures from passages 7–11 were selected for experimental use.

Human endothelial cells (HUVEC) were isolated from human umbilical veins and harvested by enzymatic treatment with chymotrypsin. HUVEC were grown in Medium 199 (M199; Biozol, Munich, Germany), supplemented with 10% FCS, 10% pooled human serum, 20 μg/ml endothelial cell growth factor (Boehringer, Mannheim, Germany), 0.1% heparin, 100 ng/ml gentamycin and 20 mM HEPES-buffer (pH 7.4). Subcultures from passages 2–6 were selected for experimental use.

### Reagents

Valproic acid was a gift from G. L. Pharma GmbH, Lannach, Austria. Everolimus was provided by Novartis Pharma AG, Basel, Switzerland. IFN-alpha-2a (IFNα) was obtained from Roche Diagnostics GmbH, Mannheim, Germany. VPA was used at a final concentration of 1 mM. Everolimus was dissolved in Dimethylsulfoxid (DMSO) as a 10 mM stock solution and stored in aliquots at −20°C. Prior to experiments, everolimus was diluted in cell culture medium. The final concentration of IFNα was 200 U/ml, if not otherwise indicated. The PCa cells were treated either with VPA, everolimus or with IFNα, or with a combination of the three agents for 3 days [triple drug (TD)]. Controls remained untreated. To exclude toxic drug effects, cell viability was determined by trypan blue (Gibco/Invitrogen). For apoptosis detection the expression of Annexin V/propidium iodide (PI) was evaluated using the Annexin V-FITC Apoptosis Detection kit (BD Pharmingen, Heidelberg, Germany). Tumour cells were washed twice with PBS, and then incubated with 5 μl of Annexin V-FITC and 5 μl of PI in the dark for 15 min. at RT. Cells were analysed on a FACScalibur (BD Biosciences, Heidelberg, Germany). The percentage of apoptotic cells (early and late) in each quadrant was calculated using CellQuest software (BD Biosciences).

### Tumour cell growth

Cell growth was assessed using the 3-(4,5-dimethylthiazol-2-yl)-2,5-diphenyltetrazolium bromide (MTT) dye reduction assay (Roche Diagnostics, Penzberg, Germany). Treated *versus* non-treated PC-3, DU-145 or LNCaP cells (100 μl, 1 × 10^4^ cells/ml) were seeded onto 96-well tissue culture plates. After 24, 48 and 72 hrs, MTT (0.5 mg/ml) was added for an additional 4 hrs. Thereafter, cells were lysed in a buffer containing 10% SDS in 0.01 M HCl. The plates were allowed to stand overnight at 37°C, 5% CO_2_. Absorbance at 570 nm was determined for each well using a microplate ELISA reader. Each experiment was done in triplicate. After subtracting background absorbance, results were expressed as mean cell number.

### Cell cycle analysis

Tumour cells were grown to 70% confluency and then treated with either VPA, everolimus, IFNα or with TD (controls remained untreated). Cell cycle analysis was carried out after 24 hrs. After 24 hrs tumour cell populations were stained with PI using a Cycle TEST PLUS DNA Reagent Kit (BD Pharmingen) and then subjected to flow cytometry with a FACScan flow cytometer (BD Biosciences). 10,000 events were collected from each sample. Data acquisition was carried out using Cell-Quest software and cell cycle distribution calculated with the ModFit software (BD Biosciences). The number of gated cells in G1, G2/M or S-phase was presented as %.

### Tumour cell adhesion to an endothelial monolayer

To analyse tumour cell adhesion, HUVEC were transferred to 6-well multiplates (Falcon Primaria; BD Biosciences) in complete HUVEC-medium. When confluency was reached, treated *versus* non-treated PC-3, DU-145 and LNCaP cells were detached from the culture flasks by accutase (PAA Laboratories, Cölbe, Germany) and 0.5 × 10^6^ cells were then added to the HUVEC monolayer for 1, 2 or 4 hrs. Subsequently, non-adherent tumour cells were washed off using warmed (37°C) Medium 199. The remaining cells were fixed with 1% glutaraldehyde. Adherent tumour cells were counted in five different fields of a defined size (5 × 0.25 mm^2^), using a phase contrast microscope, and the mean cellular adhesion rate was calculated.

### Attachment to extracellular matrix components

6-well plates were coated with collagen G (extracted from calfskin, consisting of 90% collagen type I and 10% collagen type III; Seromed; diluted to 400 μg/ml in PBS), laminin (derived from the Engelbreth–Holm–Swarm mouse tumour; BD Biosciences; diluted to 50 μg/ml in PBS), or fibronectin (derived from human plasma; BD Biosciences; diluted to 50 μg/ml in PBS) overnight. Culture plates treated with Poly-d-Lysin (Nunc, Wiesbaden, Germany) served to determine unspecific cell binding. Plastic dishes served as the background control. Plates were washed with 1% bovine serum albumin (BSA) in PBS for 60 min. to block nonspecific cell adhesion. Thereafter, 0.5 × 10^6^ tumour cells were added to each well for 60 min. Subsequently, non-adherent tumour cells were washed off, the remaining adherent cells were fixed with 1% glutaraldehyde and counted under the microscope. The mean cellular adhesion rate, defined by adherent cells_coated well_ − adherent cells_background_, was calculated from five different observation fields.

### Migration and invasion assay

Serum induced cell migration and invasion were examined using 6-well Transwell chambers (Greiner, Frickenhausen, Germany) with 8-μm pores. 0.5 × 10^6^ cells/ml were incubated with either VPA, everolimus, IFNα or with TD (controls remained untreated). To evaluate cell migration, cells were then placed in the upper chamber for 20 hrs in serum-free medium without drugs. The lower chamber contained 10% serum. After incubation, the upper surface of the Transwell membrane was wiped gently with a cotton swab to remove non-migrating cells. Cells migrating to the lower surface of the membrane were stained using hematoxylin and counted. Cells migrating into the lower chamber were counted separately under the microscope. To evaluate cell invasion towards the serum gradient, Transwell chambers were coated with collagen (400 μg/ml). Treated *versus* non-treated PCa cells were then added and the number of cells migrating to the lower membrane surface or into the lower compartment was quantified. Graphical results are shown as % inhibition, compared to the 100% untreated control.

### Integrin surface expression

Tumour cells were washed in blocking solution (PBS, 0.5% BSA) and then incubated for 60 min. at 40°C with phycoerythrin (PE)-conjugated monoclonal antibodies directed against the following integrin subtypes: Anti-α1 (IgG1; clone SR84), anti-α2 (IgG2a; clone 12F1-H6), anti-α3 (IgG1; clone C3II.1), anti-α4 (IgG1; clone 9F10), anti-α5 (IgG1; clone IIA1), anti-α6 (IgG2a; clone GoH3), anti-β1 (IgG1; clone MAR4), anti-β3 (IgG1; clone VI-PL2) or anti-β4 (IgG2a; clone 439–9B; all: BD Pharmingen). Integrin expression of tumour cells was then measured using a FACscan (BD Biosciences; FL-2H (log) channel histogram analysis (1 × 10^4^ cells/scan) and expressed as mean fluorescence units. A mouse IgG1-PE (MOPC-21) or IgG2a-PE (G155–178; all: BD Biosciences) was used as an isotype control.

### Real time qPCR

RT qPCR was also done in triplicate. cDNA-synthesis was performed using 3 μg of total RNA per sample, according to the manufacturer’s protocol by AffinityScript QPCR cDNA Synthesis Kit (Stratagene, Amsterdam, The Netherlands). Quantitative gene expression analysis by Real Time PCR was performed by the Mx3005p (Stratagene), using SYBR-Green SuperArray (SABioscience Corporation, Frederick, MD, USA) and SuperArray primer sets: GAPDH (NM_002046.3, Hs.592355), integrin α1 (ITGA1, NM_181501, Hs.644352), integrin α2 (ITGA2, NM_002203, Hs.482077), integrin α3 (ITGA3, NM_002204, Hs.265829), integrin α5 (ITGA5, NM_002205, Hs.505654), integrin α6 (ITGA6, NM_000210, Hs.133397), integrin β1 (ITGB1, NM_002211, Hs.643813), integrin β3 (ITGB3, NM_000212, Hs.218040), integrin β4 (ITGB4, NM_000213, Hs.632226; all: SABioscience Corporation). Calculation of the relative expression of each gene was done by the ΔΔCt method in the analysis program of SABioscience Corporation. The housekeeping gene GAPDH was used for normalisation.

### Western blot analysis

To explore cell cycle regulating proteins, as well as the integrin protein level, tumour cell lysates were applied to a 7% polyacrylamide gel and electrophoresed for 90 min. at 100 V. The protein was then transferred to nitrocellulose membranes. After blocking with non-fat dry milk for 1 hr, the membranes were incubated overnight with monoclonal antibodies directed against cell cycle proteins: Cdk1 (IgG1, clone 1), cdk2 (IgG2a, clone 55), cdk4 (IgG1, clone 97), cyclin B (IgG1, clone 18), cyclin D1 (IgG1, clone G124-326), cyclin E (IgG1, clone HE12), p21 (IgG1, clone 2G12), p27 (IgG1, clone 57), retinoblastoma protein (Rb; IgG1, clone XZ55; all: BD Pharmingen). Integrins were analysed using the monoclonal antibodies listed above. HRP-conjugated goat-anti-mouse IgG (Upstate Biotechnology, Lake Placid, NY, USA; dilution 1:5000) served as the secondary antibody. The membranes were briefly incubated with ECL detection reagent (ECL™, Amersham/GE Healthcare, München, Germany) to visualize the proteins and exposed to an x-ray-film (Hyperfilm™ EC™, Amersham/GE Healthcare). β-actin (1:1000; Sigma-Aldrich, Taufenkirchen, Germany) served as the internal control.

To evaluate cell signalling, PCa cells were treated with either VPA, everolimus, IFNα or with the TD combination. They were then left for 2 hrs in serum-free cell culture medium and subsequently stimulated for 30 min. with epidermal growth factor (EGF; 100 ng/ml) to activate the cell signalling cascade 9. The following monoclonal antibodies were used: Akt (IgG1, clone 55, dilution 1:500), phospho Akt (pAkt; IgG1, clone 104A282, pSer473, dilution 1:500), EGFr (IgG1, clone 13/EGFR, dilution 1:500), phospho EGFr (pEGFr; IgG1, clone 74, dilution 1:1000), ERK1 (IgG1, clone MK12, dilution 1:5000), ERK2 (IgG2b, clone 33, dilution 1:5000), phospho ERK1/2 (pERK1/2; IgG1, clone 20A, pT202/pY204, dilution 1:1000; all: BD Biosciences), p70S6k (IgG, clone 49D7, dilution 1:1000), phospho p70S6k (pp70S6k; IgG, clone 108D2, Thr389, dilution 1:1000; all: New England Biolabs, Frankfurt, Germany). To investigate histone acetylation, tumour cells were treated with the drugs for 24 hrs and cell lysates were marked with anti-acetylated H3 (IgG, clone Y28, dilution 1:500) or anti-acetylated H4 (Lys8, polyclonal IgG, dilution 1:500; all from Biomol GmbH, Hamburg, Germany). Analysis was performed by western blot.

### Statistics

All experiments were performed 3–6 times. Statistical significance was determined by the Wilcoxon–Mann–Whitney *U*-test. Differences were considered statistically significant at a *P*-value less than 0.05.

## Results

### PC-3, DU-145 and LNCaP cell growth and adhesion to HUVEC

**Figure 1:**
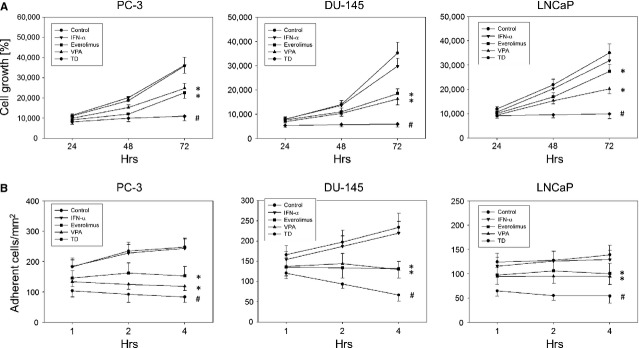
(A) Cell growth analysis of PC-3, DU-145 and LNCaP cells. Tumour cells were treated with either 100 U/ml IFNα, 1 nM everolimus or 1 mM VPA, or with all compounds simultaneously (TD). Controls remained untreated. Cells were counted after 24, 48 and 72 hrs. One representative experiment of six is shown. * indicates significant difference to controls, # indicates significant difference to single drug treatment. (B) Adhesion of prostate cancer cells to HUVEC. PC-3, DU-145 and LNCaP cells were treated with 100 U/ml IFNα, 1 nM everolimus or 1 mM VPA, or with all compounds simultaneously (TD). Controls remained untreated. Tumour cells were added at a density of 0.5 × 10^6^ cells/well to HUVEC monolayers for 1, 2 or 4 hrs. Non-adherent tumour cells were washed off and the remaining cells fixed and counted in five different fields (5 × 0.25 mm^2^). Mean values were calculated from five counts. One representative of six experiments is shown. * indicates significant difference to controls, ^#^ indicates significant difference to single drug treatment.

Valproic acid and everolimus significantly inhibited the growth of PC-3, DU-145 and LNCaP cells, whereas IFNα exerted no significant decrease in cell growth in any cell line (Fig.1A). TD application resulted in nearly complete inhibition of tumour cell growth in all three cell lines.

Valproic acid and everolimus significantly diminished the binding capacity of tumour cells on HUVEC in all three cell lines, whereas no effect was exerted by IFNα alone (Fig.1B). TD resulted in a significantly greater decrease in tumour cell attachment than any single drug.

Since everolimus and VPA distinctly inhibited tumour cell growth and adhesion to HUVEC in all cell lines, the PC-3 cell line was randomly chosen for all further investigations concerning cell cycle progression, adhesion, migration, integrin expression and intracellular signalling.

### PC-3 cell cycle progression and associated proteins

**Figure 2:**
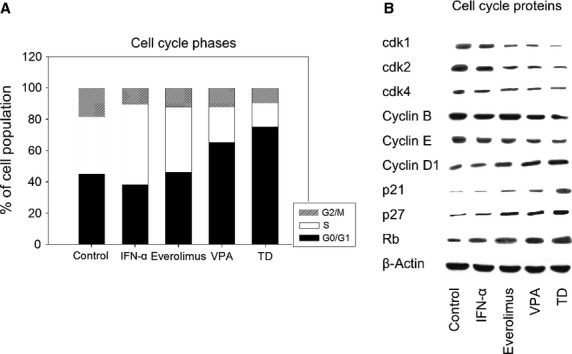
(A) Cell cycle analysis of PC-3 cells. Tumour cells were treated either with 100 U/ml IFNα, 1 nM everolimus or 1 mM VPA, or with all compounds simultaneously (TD). Controls remained untreated. Cell cycle analysis was carried out after 24 hrs. The cell population at each checkpoint is expressed as percentage of total analysed cells. One representative experiment of three is shown. (B) Western blot of cell cycle proteins. PC-3 cells were treated either with 100 U/ml IFNα, 1 nM everolimus or 1 mM VPA, or with all compounds simultaneously (TD). Controls remained untreated. β-actin served as the internal control. The figure shows one representative from three separate experiments.

Valproic acid decreased the G2/M- and S-phase and distinctly augmented the G0/G1-phase cells, compared to control (Fig.2A). Everolimus also reduced the G2/M-phase, but increased the number of S-phase cells. IFNα enhanced the S-phase and reduced the G2/M- and G0/G1-phase. Application of TD caused more cells to remain in the G0/G1-phase and fewer in the S- phases compared to any single drug. The Annexin V-FITC-assay showed no increase in apoptotic events with any of the drugs alone or during the triple application (data not shown).

The expression of cell cycle proteins was differently modified compared to control, depending on the applied drug (Fig.2B). VPA reduced cdk1, cdk2, cdk4, cyclin B and cyclin E while cyclin D1, p21, p27 and Rb were increased. On the whole, everolimus showed similar effects except for the expression of cyclin B where no alteration was observed. IFNα stimulated the expression of Rb. TD reduced the expression of cdk1, cdk2, cdk4, cyclin B and cyclin E and augmented that of cyclin D1, p21, p27 and Rb to a greater extent than any drug alone.

### PC-3 cell adhesion to extracellular matrix, migration and invasion

**Figure 3:**
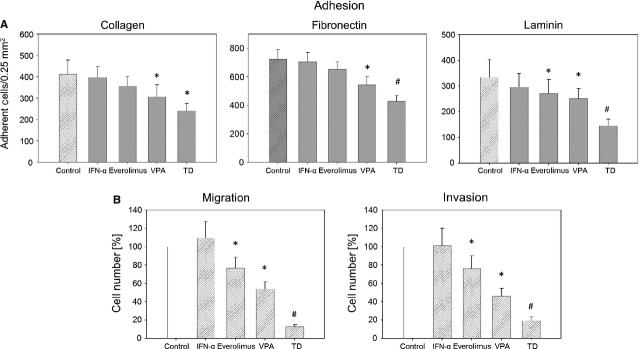
(A) Adhesion of prostate cancer cells to extracellular matrix proteins. PC-3 cells were treated with 100 U/ml IFNα, 1 nM everolimus or 1 mM VPA, or with all compounds simultaneously (TD). Cells were added to immobilized collagen, laminin or fibronectin at a density of 0.5 × 10^6^ cells/well for 60 min. Plastic dishes were used to evaluate unspecific binding (background control). One representative of six experiments is shown. * indicates significant difference to controls, ^#^ indicates significant difference to single drug treatment. (B) PC-3 cell migration (left) and invasion (right). Cells treated with 100 U/ml IFNα, 1 nM everolimus or 1 mM VPA, or with all compounds simultaneously (TD). Controls were set to 100%. * = significant difference to controls. # = significant difference to single drug treatment.

Cell adhesion to the extracellular matrix proteins, collagen, fibronectin and laminin, is shown in Figure3A. IFNα exerted no effect on tumour cell adhesion. Whereas everolimus blocked cellular attachment only to laminin, VPA inhibited adhesion to all three matrix proteins. TD further diminished attachment to fibronectin and laminin. Its effect on collagen was similar to that of VPA.

Compared to controls, VPA and everolimus diminished migration and invasion of the tumour cells (Fig.3B), whereas IFNα was ineffective. TD decreased both migration and invasion capacity much more effectively than any single agent.

### PC-3 integrin subtype expression

**Figure 4:**
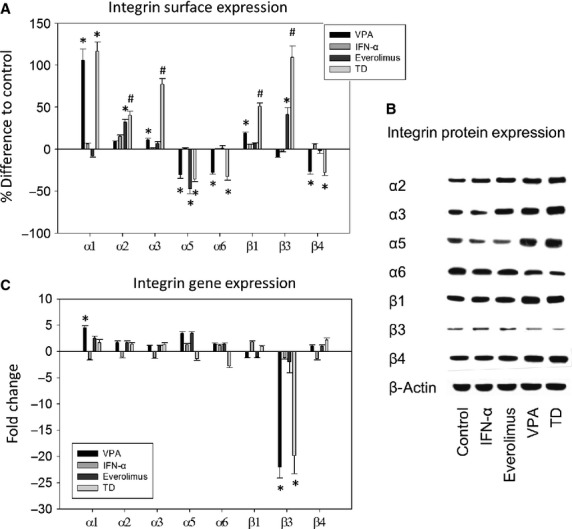
(A) Integrin expression on cell surface. PC-3 cells were treated with 100 U/ml IFNα, 1 nM everolimus or 1 mM VPA, or with all compounds simultaneously (TD). Controls remained untreated. Mean fluorescence units are given in percentage difference to the controls. One of three independent experiments is shown. * indicates significant difference to controls, # indicates significant difference to single drug treatment. (B) Western blot analysis of integrin protein expression. PC-3 cells were treated either with 100 U/ml IFNα, 1 nM everolimus or 1 mM VPA, or with all compounds simultaneously (TD). Controls remained untreated. β-actin served as the internal control. The figure shows one representative from three separate experiments. (C) Integrin gene expression. PC-3 cells were treated with 100 U/ml IFNα, 1 nM everolimus or 1 mM VPA, or with all compounds simultaneously (TD). Controls remained untreated. One representative from three separate experiments is shown. * indicates significant difference to controls, ^#^ indicates significant difference to single drug treatment (*i.e*. to VPA, IFNα or everolimus).

Interferon alpha exerted no effect on integrin surface expression, whereas VPA enhanced the expression of α1, α3 and β1 and reduced the level of α5, α6 and β4 (Fig.4A). Integrin α4 expression was not detected on the surface of untreated cells (data not shown). Everolimus increased the expression of α2 and β3 and reduced the level of α5. Compared to IFNα, VPA or everolimus alone, when exposed to TD cancer cells demonstrated augmented expression of α2, α3, β1 and β3.

Figure4B demonstrates that IFNα moderately up-regulated α2 in the intracellular integrin protein content. Everolimus down-regulated α5 and up-regulated α2, α3 and β3 (Fig.4B). VPA enhanced the expression of α2, α3, α5, β1 and β4 and diminished α6 and β3. TD exerted the same effects as VPA, but they were more intensified.

Valproic acid distinctly increased the α1 coding mRNA and pronouncedly down-regulated the β3 gene transcriptional activity (Fig.4C). The effect on β3 exerted by VPA was also observed when TD was applied. IFNα and everolimus did not act on the integrin coding mRNA in PC-3 cells. α4 coding mRNA was not detected, regardless of the treatment protocol (data not shown).

### PC-3 intracellular signalling

**Figure 5:**
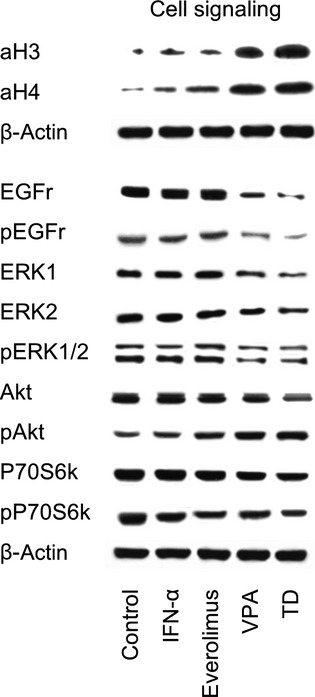
Western blot analysis of cell signalling proteins. PC-3, DU-145 or LNCaP cells were treated with 100 U/ml IFNα, 1 nM everolimus or 1 mM VPA, or with all compounds simultaneously (TD). Controls remained untreated. Cells were kept for 2 hrs in serum-free cell culture medium and subsequently stimulated for 30 min. with EGF (100 ng/ml). β-actin served as the internal control. The figure shows one representative from three separate experiments.

Valproic acid and TD both strongly augmented the acetylation of histone 3 (aH3) and histone 4 (aH4) (Fig.5). VPA decreased EGFr (total and activated), ERK1, ERK2, pERK and pP70S6k. pAkt was enhanced. Everolimus reduced phosphorylation of P70S6k. IFNα did not exert an effect on these cell signalling proteins. Compared to the monotherapies, treatment with TD displayed an intensified decrease in EGFr (total and activated), ERK1, ERK2 and P70S6k. Simultaneously, the combination treatment induced augmented acetylation of H3.

## Discussion

Novel targeted approaches have revolutionized therapy for metastatic and castration-resistant PCa 12. However, despite selective inhibition of critical molecular elements in the neoplastic machinery instigating a significant improvement in survival, no cure for disseminated cancer has been achieved and drug resistance inevitably develops during therapy 13. Drug resistance depends on tumour and treatment type and often involves a multitude of cellular processes responsible for the decreased response to the anticancer agent 14,15. One option to combat acquired drug resistance is to combine several drugs targeting different pathways. Such combinations can inhibit reciprocal loops and/or compensatory rewiring and increase efficacy 16. Growing evidence points to a crucial role of histone modification, particularly acetylation and deacetylation, as significant epigenetic mechanisms of gene regulation, which play essential roles in oncogenesis and progression of PCa 9,17,18. Recently, it has been reported that combining HDAC-blockade with either low dosed IFNα 9 or mTOR-inhibitors 10,11 potentiates antineoplastic effects. On this basis a comparative assessment of the malignant behaviour of PCa cells treated with combined HDAC-blockade, mTOR-inhibitor and low dosed IFNα, as opposed to each single agent, was performed.

Simultaneous block of HDAC and mTOR related signalling in conjunction with low dosed IFNα led to distinct inhibition of cell growth and adhesion capacity to endothelium in PC-3, DU-145 and LNCaP cells, and was intensified compared to single agent application. Despite IFNα’s documented anti-proliferative influence in some types of cancer 19, alone it yielded no noteworthy effect on cell growth or attachment to HUVEC in any of the employed PCa cell lines. Cinatl *et al*. 20 also observed no appreciable impact of IFNα as a monotherapy on growth and attachment rate to endothelium in neuroblastoma cells, but profound effects when combined with VPA. Kuljaca *et al*. 21 have also reported co-operative cytotoxic and antiangiogenic potentiation of HDAC-inhibition with IFNα in a range of cancer cell lines including DU-145 and LNCaP cells, whereas IFNα alone was not effective. IFNα, thus seems rather to act as a tumour cell sensitizer for treatment with other agents.

Triple drug promoted a distinct increase in the G0/G1 phase and diminished the G2/M and S-phases. The diminished S-phase and increased G0/G1 effect went much beyond that of single drug regimens and might contribute to the significantly decreased cell growth, which was observed. In accordance with the differential impact on cell cycle progression, each single drug modified the cell cycle proteins differently. Whilst IFNα was inefficient in altering protein expression, everolimus and VPA both reduced cdk1, cdk2, cdk4, cyclin B (only VPA) and cyclin E and up-regulated the expression of cyclin D1, p21, p27 and Rb. Pronounced combinatory effects were observed for the TD regimen. Since the cdk-cyclin-Rb-axis acts as a shifting mechanism in cell cycle entry and progression, alteration of these elements is of particular significance. As reported by Rigas *et al*. 22, treating several androgen-independent PCa cell lines including PC3 with a cdk1/cdk2-inhibitor led to a profound decrease in cell growth and G1 cell cycle phase arrest. Comstock *et al*. 23 presented evidence that selective blocking of cdk4/6 significantly impaired the capacity of PCa cells to proliferate by promoting a robust G1-arrest and induced growth inhibition of PCa xenografts. In the present investigation, TD exerted a major impact on G1-S-transition, which was accentuated by a significant down-regulation of cyclin E and its catalytic partner cdk2. This mechanism in the late G1 phase mediates hyper-phosphorylation and inactivation of Rb as well as E2F release, both required for S-phase transition 23,24. Deregulation of cyclin E expression is a well-known process in different cancer types and might represent a therapeutic target 25. Thus, in cyclin E-over-expressing hepatocellular carcinoma, pharmacological down-regulation of this protein has been shown to initiate cell apoptosis, inhibit proliferation and block cell growth *in vitro* 26. Another important effect of TD was to reduce cyclin B and its functional complex with cdk1, essential for promoting M phase progression. Knocking down the cdk1-cyclin B complex in everolimus-resistant PC3 cells has been shown to decrease cell growth activity and partially abolish drug resistance 27. In addition, an increase in p21 and p27 expression delineates further important antineoplastic processes, since their therapeutic up-regulation has been associated with PCa prevention 28.

In the present investigation, treating cells with TD provoked an enhancement of Rb and attenuation of cdk4 activity, pointing to inhibition of the early G1 phase entry. The Rb antibody we used did not discriminate between the phosphorylated and non-phosphorylated form. Still, up-regulation of Rb by dual targeting of the Akt and mTOR signalling pathways has recently been demonstrated to be a highly effective option for inhibiting PCa 29. It is difficult to interpret the elevation of cyclin D1 under combination therapy. As recently reported, cyclin D/cdk4 complex mono-phosphorylates Rb and inactivates its G0 function, preventing cell cycle exit 30. Cyclin D1 is only in part typically associated with cdk4 31 and has multiple cdk4-independent functions as a transcriptional co-repressor 32. Contradictory results have been reported on the role of cyclin D in PCa 33. Inhibition of cyclin D1 blocked growth factor-induced cell cycle progression in LNCaP cells 34 while cyclin D1 over-expression in LNCaP cells enhanced S-phase entry, increased colony formation and promoted resistance to androgen ablation 35. In contrast, transfection of a fragment of cyclin D1 encoding (repressor domain) inhibited DNA synthesis in LNCaP cells 32. Speculatively, augmentation of cyclin D1 might trigger a negative feedback loop, cancelling the blockade of tumour cells in reaching late G1 restriction. This could induce downstream cyclin activation for cell cycle progression. Alternatively, it might reflect enhanced transcriptional blockade of genes acting in PCa tumourigenesis and therefore exert an instantaneous antineoplastic effect.

Triple drug exhibited an inhibitory effect on systemic dissemination, a crucial facet in PCa oncogenesis. Dissemination entailing reduced attachment to extracellular matrix elements, migration and invasion activity of PC3 cells converts a curatively treatable condition into a fatal disease. Whilst each component of the therapeutic compound altered integrin expression differentially, the combined regimen mediated a more pronounced increase of α2, α3, β1 and β3 on the cell membrane than each monotherapy. Additionally, surface expression of α5, α6 and β4 in the presence of TD was down-regulated compared to controls, whereas only the α6 type showed a reduction in total integrin content. An interesting aspect is associated with the β3 integrin, being up-regulated on the cell surface, while the integrin β3 message was down-regulated. The opposite behaviour, which is not uncommon 36, could mean that a high surface presence initiates feedback loops leading to reduced *de novo* synthesis.

The functional role of integrins in tumourigenesis entails more than that of a mechanistic receptor for cell-cell and cell-matrix attachment 36. Integrin stimulation activates a number of intracellular signalling pathways involved in cell proliferation, differentiation, motility and other essential cell functions 37 and may not always directly correlate with malignant behaviour. Zutter *et al*. 38 observed a significantly decreased α2 and β1 expression in poorly differentiated adenocarcinomas of the breast, compared to normal tissue. Ramirez *et al*. 39 has reported that the integrin subunits α2 and β1 act as metastatic suppressors and loss of α2 is associated with impaired survival in breast and PCa. An increase in β1 changed a malignant phenotype to a less invasive one 40. Therefore, elevation of the α2 and β1 cell surface levels observed in our study might explain a mechanism by which TD inhibits cell dissemination. A study on PCa stem cells 41 provided evidence that α1 enhanced homing and differentiation. Blocking this integrin diminished the rate of cells expressing PCa markers. In this context, up-regulation of α1 might play a role in hindering cell dedifferentiation and decreasing aggressive behaviour.

Triple drug most profoundly deactivated p70S6k compared to each drug applied alone, at least in part contributing to significant cell growth suppression observed with this regimen. Consistent with this data, pharmacologically induced activation blockade of p70S6k was reportedly associated with suppression of cell proliferation and induction of apoptosis in PC-3 cells 42,43. Surprisingly, Akt phosphorylation was also considerably stimulated under the combined regimen. In line with this, Sun *et al*. 44 observed a down-regulation of p70S6k activity paradoxically combined with increased Akt phosphorylation in lung cancer cells. Most groups have described activation of Akt signalling with respect to PCa progression in cell lines and tissue specimens 45,46. However, this pathway is complex and includes multiple controlling and feedback mechanisms. Evidence provided by Bjerke *et al*. show that activation of Akt signalling induces a TGFβ-mediated restraint on PCa progression and metastasis 47. Total Akt was massively reduced under TD, implying that less Akt may still be able to generate Akt related signals in the form of increased pAkt, which was observed in the present investigation. Whether elevated Akt phosphorylation contributes to anticancer effects or embodies a compensatory feedback mechanism of the neoplastic machinery remains to be determined.

## Conclusions

The triple application of VPA, everolimus and low dosed IFNα exerted significantly superior blockage of tumour cell growth and dissemination potential, compared with that achieved by any single agent. Combining HDAC- and mTOR-inhibition with interferon alpha should be considered when planning clinical trials to counteract resistance development and improve outcome in PCa patients.

## Conflicts of interest

The authors declare that they have no competing interests.
